# Crystal structure of poly[[(μ_3_-hydroxido-κ^3^ *O*:*O*:*O*)(μ_3_-selenato-κ^3^ *O* ^1^:*O* ^2^:*O* ^3^)tris­[μ_3_-2-(1,2,4-triazol-4-yl)acetato-κ^3^ *N* ^1^:*N* ^2^:*O*]tricopper(II)] dihydrate]

**DOI:** 10.1107/S2056989019009812

**Published:** 2019-07-16

**Authors:** Kostiantyn V. Domasevitch, Andrey B. Lysenko

**Affiliations:** a Inorganic Chemistry Department, Taras Shevchenko National University of Kyiv, Volodimirska Street 64, Kyiv 01033, Ukraine

**Keywords:** crystal structure, metal-organic frameworks, secondary building units, copper(II) complexes

## Abstract

The title coordination polymer, {[Cu_3_(C_4_H_4_N_3_O_9_)_3_(SeO_4_)(OH)]·2H_2_O}_*n*_ or ([Cu_3_(μ_3_-OH)(trgly)_3_(SeO_4_)]·2H_2_O), crystallizes in the monoclinic space group *P*2_1_/*c*. The three independent Cu^2+^ cations adopt distorted square-pyramidal geometries with {O_2_N_2_+O} polyhedra. The three copper centres are bridged by a μ_3_-OH anion, leading to a triangular [Cu_3_(μ_3_-OH)] core. 2-(1,2,4-Triazol-4-yl)acetic acid (trgly-H) acts in a deprotonated form as a μ_3_-κ^3^ *N* ^1^:*N* ^2^:*O* ligand. The three triazolyl groups bridge three copper centres of the hydroxo-cluster in an *N* ^1^:*N* ^2^ mode, thus supporting the triangular geometry. The [Cu_3_(μ_3_-OH)(tr)_3_] clusters serve as secondary building units (SBUs). Each SBU can be regarded as a six-connected node, which is linked to six neighbouring triangles through carboxyl­ate groups, generating a two-dimensional uninodal (3,6) coordination network. The selenate anion is bound in a μ_3_-κ^3^ *O* ^1^:*O* ^2^:*O* ^3^ fashion to the trinuclear copper platform. The [Cu_3_(OH)(trgly)_3_(SeO_4_)] coordination layers and guest water mol­ecules are linked together by numerous O—H⋯O and C—H⋯O hydrogen bonds, leading to a three-dimensional structure.

## Abstract 2

The self-assembly of Cu^II^ selenate and 1,2,4-triazol-4-yl-acetic acid (trgly-H) in aqueous solution under hydro­thermal conditions affords a two-dimensional coordination network ([Cu_3_(μ_3_-OH)(trgly)_3_(SeO_4_)]·2H_2_O) based on triangular coordination clusters [Cu_3_(μ_3_-OH)] as secondary building units (SBUs). The trinuclear motif is supported by three [N—N] triazole bridges and a facially coordinating tripodal SeO_4_^2−^ anion. This results in a less distorted square-pyramidal arrangement around the five-coordinate copper(II) centres in comparison to that of the analogous isomorphous Cu_3_(μ_3_-OH)(trgly)_3_(SO_4_)]·2H_2_O complex.

## Chemical context   

Extended coordination networks incorporating trinuclear 1,2,4-triazole (tr)-based hydroxo(oxo) clusters [Cu_3_(μ_3_-OH/or O)(tr)_3_] as secondary building units (SBUs) are a subject of high inter­est in many inter­disciplinary fields including gas storage and sorption (Lincke *et al.*, 2012 ▸), magnetism (Ouellette *et al.*, 2006 ▸), anion exchange and separation (Wang *et al.*, 2007 ▸). In these clusters, the copper(II) cations display either distorted tetra­gonal–pyramidal (TP) or (and) octa­hedral coordination geometries, two of the most stable configurations in the OH^−^/tr ligand arrangement. Typically, the basal plane for a five-coordinate Cu^II^ atom (or the equatorial plane for six-coordinate Cu^II^) consists of two nitro­gen atoms from two *trans*-coordinated tr groups, an oxygen atom from OH^−^/O^2−^ and an O (N, or Cl^−^) donor atom (or anion) from an extra ligand, whereas the apical position is occupied by a water mol­ecule or anionic ligand (Lysenko *et al.*, 2006 ▸; Naik *et al.*, 2010 ▸). The alternative trigonal–bypiramidal (TBP) environment around the copper centres can not be realized in the specific ligand configuration. Addison *et al.* (1984 ▸) introduced a useful structural parameter, τ, as a criterion for distinguishing between TP and TBP polyhedra. This parameter, which varies from 0 (in TP) to 1 (in TBP), could perhaps be used to predict the anion binding affinity of closely related anions (*e.g.* SO_4_^2−^ *versus* SeO_4_^2−^) toward the [Cu_3_(μ_3_-OH/or O)(tr)_3_] SBUs. The higher binding affinity might be associated with the lower τ parameter. As a matter of fact, the [Cu_3_(μ_3_-OH/or O)(tr)_3_] cationic clusters are perfectly suited for the binding of tetra­hedral anions through its three apical sites. In this context, it would be inter­esting to clarify how the size of the coordinating anions correlates with the τ value. In this paper, we report the crystal structure of the title Cu^2+^ complex, (I), which was prepared by reacting CuSeO_4_ and trgly-H in an aqueous solution under hydro­thermal conditions. The compound is isomorphous to the [Cu_3_(μ_3_-OH)(trgly)_3_(SO_4_)]·2H_2_O complex (Vasylevs’kyy *et al.*, 2014 ▸).
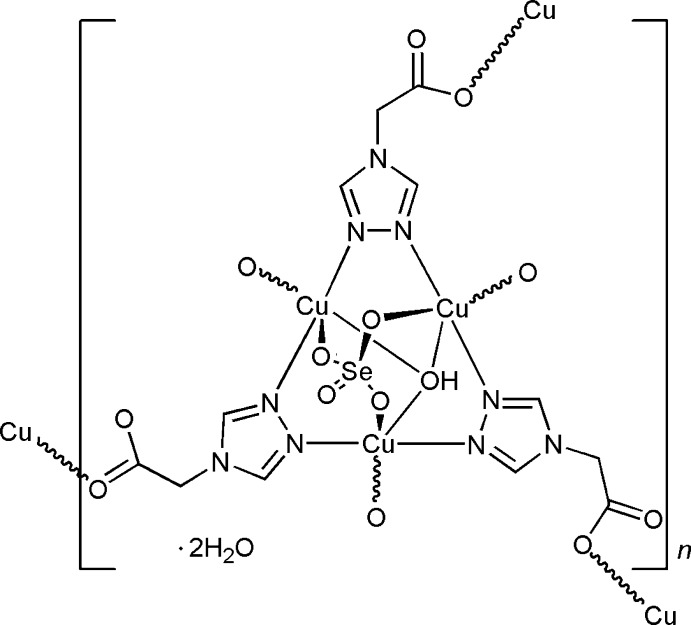


## Structural commentary   

**Figure 1:**
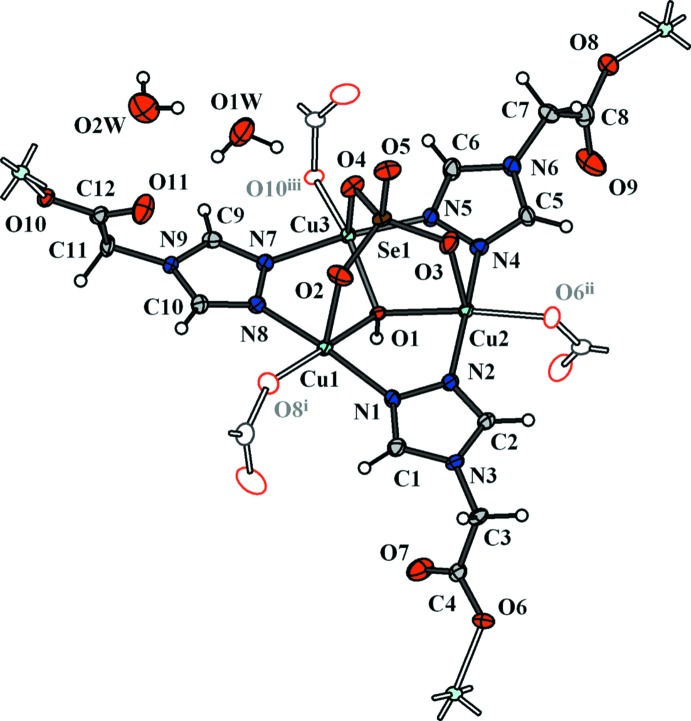
A portion of the structure of (I), showing the atom-labelling scheme and the copper coordination environments. Displacement ellipsoids are drawn at the 50% probability level. [Symmetry codes: (i) *x*, *y*, *z* − 1; (ii) *x*, −*y* − 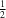, *z* + 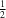; (iii) *x*, −*y* + 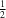, *z* + 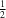].

**Figure 2:**
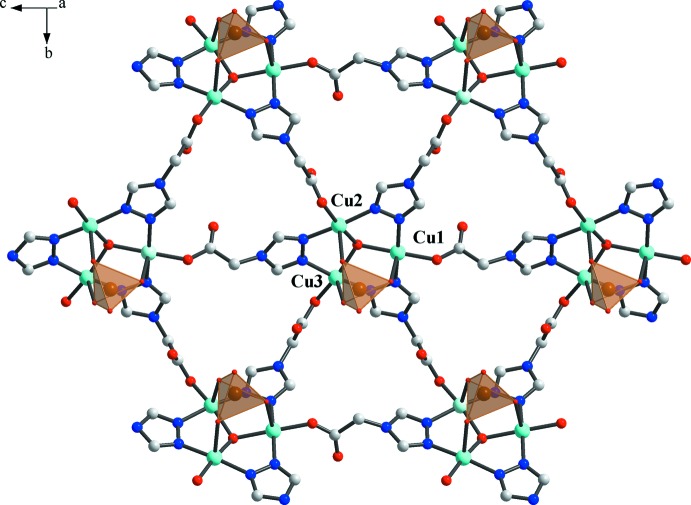
A single layer of the structure has the (3,6) topological type (view along the [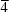
0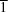] direction, selenates shown as tetra­hedra).

**Figure 3:**
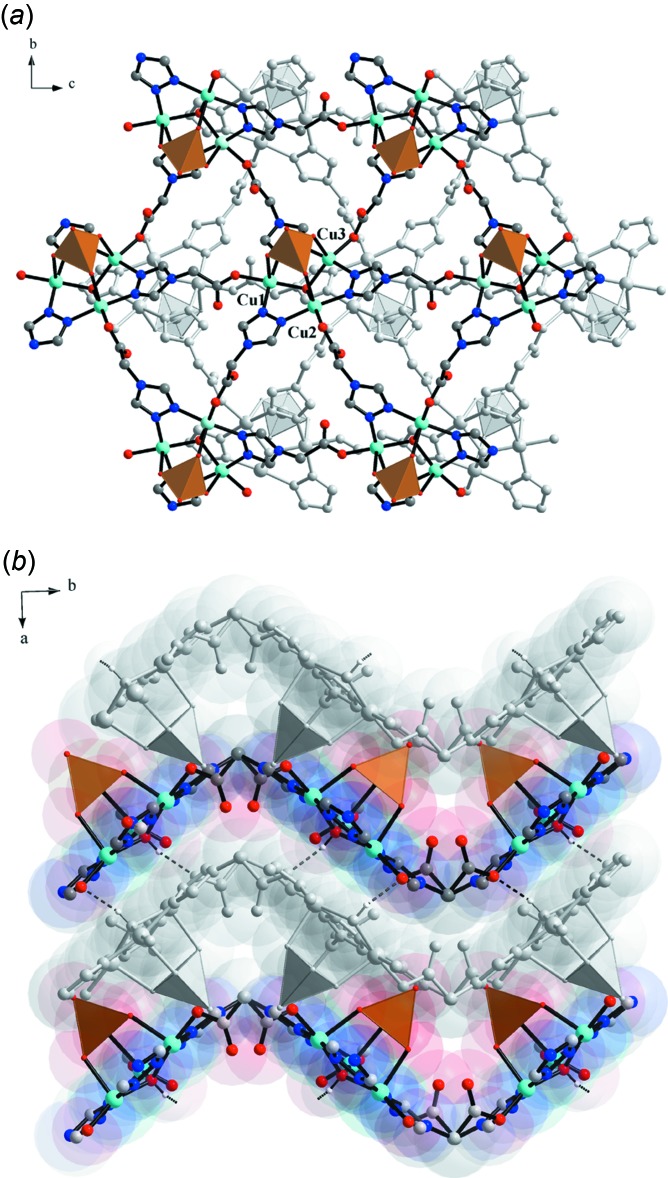
Crystal packing of compound (I) (*a*) along the *a* axis, and (*b*) along the *c* axis. In (*a*), neighboring layers are shifted relative to one another while in (*b*) they are held together by O—H⋯O hydrogen bonds between hydroxide oxygen atoms and carboxyl­ate group oxygen atoms [O1⋯O10^iv^ = 2.811 (4) Å, O1—H1*O*⋯O10^iv^ = 156°; symmetry code: (iv) −*x* + 1, *y* − 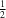, −*z* + 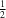].

The title compound crystallizes in the centrosymmetric monoclinic space group *P*2_1_/*c*. The asymmetric unit consists of three copper(II) cations, one selenate anion, one hydroxide anion, three deprotonated trgly^−^ ligands and two water mol­ecules (Fig. 1 ▸ and Table 1 ▸). Each copper centre adopts a similar tetra­gonal–pyramidal coordination environment with the {O_2_N_2_+O} donor set. The basal plane of Cu1 is completed by atom O1 of the *μ*_3_-bridging hydroxide group [Cu1—O1 = 2.022 (2) Å], atom N1 from one bridging tr-group [Cu1—N1 = 1.980 (3) Å], atom N8 from the other bridging tr-group [Cu1—N8, 1.993 (3) Å] and a carboxyl­ate O atom from the trgly ligand [Cu1—O8^i^ = 1.935 (3) Å; symmetry code: (i) *x*, *y*, *z* − 1]. The basal planes of Cu2 and Cu3 cations consist four short bonds as follows: Cu2—N2 = 1.979 (3), Cu2—N4 = 1.986 (3), Cu2—O1 = 2.039 (3) and Cu2—O6^ii^ 1.954 (3) Å and Cu3—N5 = 1.974 (3), Cu3—N7 = 1.982 (3), Cu3—O1 = 2.039 (2) and Cu3—O10^iii^ 1.990 (3) Å for Cu2 and for Cu3, respectively [symmetry codes: (ii) *x*, −*y* − 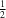, *z* + 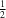; (iii) *x*, −*y* + 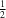, *z* + 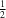]. The basal planes of the three square pyramids share a common corner at the O1 atom of the OH^−^ anion, forming a triangular [Cu_3_(μ_3_-OH)] core. The trinuclear motif is supported by a facially coordinating tripodal selenate anion [Cu1—O2 2.182 (3), Cu2—O3 = 2.146 (3) and Cu3—O4 = 2.182 (3) Å]. The value of the Addison structural parameter τ varies from 0.025 for Cu2 through 0.070 for Cu1 to 0.189 for Cu3, indicating the preference of a TP configuration (*versus* TBP) around the copper centres. A comparison of the τ values for (I) with the corresponding values for the isomorphous sulfate complex [Cu_3_(μ_3_-OH)(trgly)_3_(SO_4_)]·2H_2_O (τ = 0.021, 0.103, 0.211; Vasylevs’kyy *et al.*, 2014 ▸) indicates a lower degree of TBP distortion for the selenate compound. This tendency is also observed for the other two isomorphous MOFs [{Cu_3_(μ_3_-OH)(*X*)}_4_{Cu_2_(H_2_O)_2_}_3_(trz-ia)_12_] [*X* = SO_4_^2−^ and SeO_4_^2−^, trz-ia is the 5-(4*H*-1,2,4-triazol-4-yl)isophthalate anion], where the τ parameter values are 0.096 and 0.083 for the sulfate and selenate complexes, respectively (Lincke *et al.*, 2012 ▸). Unlike [Cu_3_(μ_3_-OH)(trgly)_3_(SO_4_)]·2H_2_O, in which the highest τ value corresponds with the longest Cu—O axial bond, the τ parameter values for the title compound do not correlate with the Cu—O axial bond lengths. Atoms Cu1 and Cu3 with the lowest and highest τ values, respectively, have the same Cu—O axial bond lengths. For compound (I), the hydroxide oxygen atom O1 is displaced by 0.532 Å from the centroid of the Cu1–Cu2–Cu3 triangular fragment, whereas for [Cu_3_(μ_3_-OH)(trgly)_3_(SO_4_)]·2H_2_O, the O–centroid distance is 0.570 Å. Thus, the larger anion–anion repulsion (OH^−^/SO_4_^2−^ *versus* OH^−^/SeO_4_^2−^) in the sulfate complex also confirms the higher TBP distortion. The trinuclear clusters function as SBUs (six-connected nodes), which self-assemble into a two-dimensional coordination network (Fig. 2 ▸) with all of the selenate anions on the same side of the coordination layer. The resultant 2D network topology can be rationalized as a (3,6) type. Inter­estingly, the selenate anions of two neighbouring layers point in opposite directions (Fig. 3 ▸).

## Supra­molecular features   

**Figure 4:**
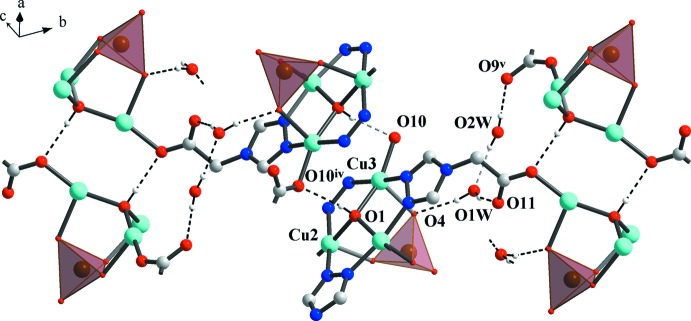
Crystal packing pattern in (I) showing the O—H⋯O hydrogen-bonding inter­actions between neighboring layers.

The trinuclear [Cu_3_(μ_3_-OH)(tr)_3_] clusters are involved in inter- and intra­molecular hydrogen-bonding inter­actions. Adjacent layers are linked together by hydrogen bonding between the hydroxide oxygen atoms (O1 as H-atom donor) and carboxyl­ate group oxygen atoms (O10 as H-atom acceptor) and are shifted with respect to each other, forming a H-bonded double layer (Fig. 3 ▸ *a*, Table 2 ▸). The guest water mol­ecules are trapped between neighboring double-layers, forming a set of hydrogen bonds to selenate oxygen atoms [O1*W*⋯O4 = 2.767 (4) Å, O1*W*—H2*W*⋯O4 = 168°], carboxyl­ate oxygen atoms [O1*W*⋯O11 = 2.940 (5) Å, O1*W*—H1*W*⋯O11 = 166°, and O2*W*⋯O9^v^ = 2.798 (5) Å, O2*W*—H3*W*⋯O9^v^ 178°, symmetry code: (v) −*x* + 1, *y* + 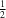, −*z* + 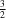] and to one another [O2*W*⋯O1*W* = 2.812 (6) Å, O2*W*—H4*W*⋯O1*W* = 159°, Fig. 4 ▸]. Apparently, the presence of the hydrogen bond between the O1*W* water mol­ecule and the selenate oxygen atom O4 leads to an increase in the trigonal–bypiramidal distortion of the square-pyramidal coordination polyhedra of Cu3 (τ = 0.189 for Cu3, markedly higher than the values of 0.070 and 0.025 for Cu1 and for Cu2, respectively) in the trinuclear [Cu_3_(μ_3_-OH)(tr)_3_] core.

**Figure 5:**
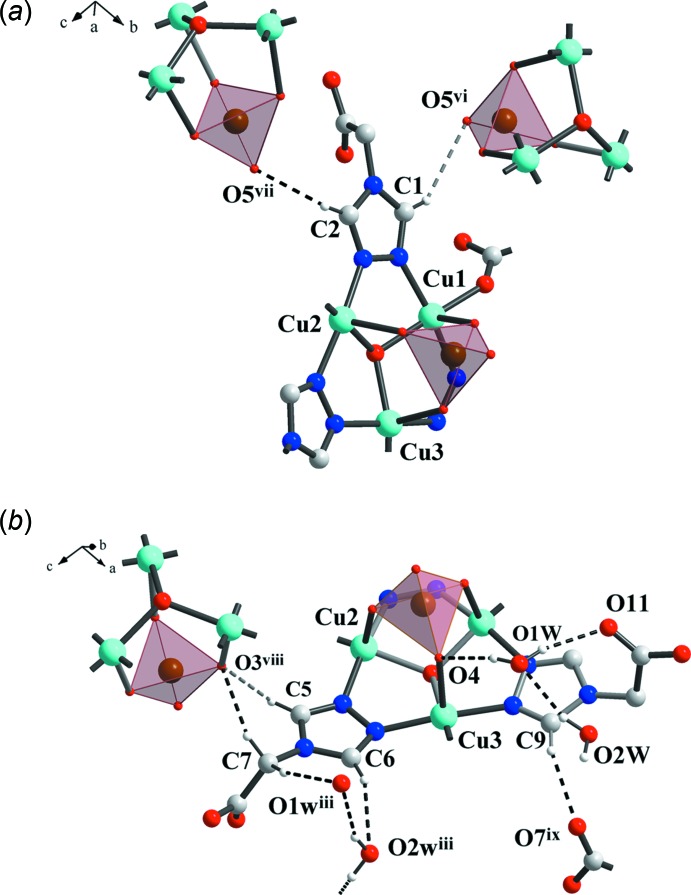
Crystal packing patterns in (I) showing the presence of C—H⋯O hydrogen bonds.

The coordination polymeric network is reinforced by weak C—H⋯O hydrogen-bonding inter­actions (Desiraju & Steiner, 1999 ▸, Fig. 5 ▸, Table 2 ▸). These C—H hydrogen bonds with one acceptor oxygen atom [C⋯O distances ranging from 2.955 (5) to 3.440 (5) Å] help to stabilize the resulting three-dimensional hydrogen-bonded network.

Thus, the hydro­thermal reaction of CuSeO_4_ and trgly-H leads to a two-dimensional coordination network [Cu_3_(μ_3_-OH)(trgly)_3_(SeO_4_)] based on the trinuclear coordination clusters [Cu_3_(μ_3_-OH)]. The five-coordinate copper(II) centres in the [Cu_3_(μ_3_-OH)(tr)_3_] SBU display less-distorted square-pyramidal arrangements in comparison to those of the isomorphous complex [Cu_3_(μ_3_-OH)(trgly)_3_(SO_4_)]·2H_2_O.

### Database survey   

Among the known [Cu_3_(μ_3_-OH/or O)(tr)_3_] complexes (CSD version 5.39, update of May 2018; Groom *et al.*, 2016 ▸), the highest possible value of τ (0.313) in the five-coordinate copper(II) cation was once observed for the copper(II)-polyoxomolybdate complex with 4-amino-1,2,4-triazole [Cu_3_(4-atrz)_3_(Mo_8_O_27_)(H_2_O)_4_]·6H_2_O (Wang *et al.*, 2015 ▸). However, the authors described the trinuclear cationic core as [Cu_3_(μ_3_-H_2_O)(4-atrz)_3_]. They also inter­preted the five-coord­inate copper geometry as trigonal–bipyramidal, although the τ parameter is closer to 0 than to 1.

## Synthesis and crystallization   

1,2,4-Triazol-4-yl-acetic acid, (trgly-H) was prepared in a yield of 30% by reacting glycine and *N*,*N*-di­methyl­formamide azine in boiling toluene under acidic conditions (Vasylevs’kyy *et al.*, 2014 ▸). Copper(II) selenate penta­hydrate was prepared by treating basic copper carbonate with selenic acid followed by crystallization. A solution of CuSeO_4_·5H_2_O (59.2 mg, 0.20 mmol) in 4 mL of water was added to a solution of trgly-H (27.2 mg, 0.20 mmol) in water (2 mL). The resulting solution was placed in a 20 mL Teflon-lined steel autoclave and heated at 393 K for 24 h. Cooling from to rt over 48 h afforded green–blue crystals of the product (yield 52%). Analysis calculated for C_12_H_17_Cu_3_N_9_O_13_Se (%): C, 18.84; H, 2.24; N, 16.48. Found: C, 18.79; H, 2.28; N, 16.40. Elemental analysis was carried out with a Vario EL-Heraeus microanalyzer.

## Refinement   

Crystal data, data collection and structure refinement details are summarized in Table 3 ▸. All C-bound H atoms were placed at calculated positions [C—H = 0.94 Å (aromatic), C—H = 0.98 Å (aliphatic)] and refined using a riding model with *U*_iso_(H) = 1.2*U*_eq_(CH). All O-bound H atoms were located in a difference-Fourier map and then fixed at O—H = 0.85 Å and with *U*_iso_(H) =1.5*U*_eq_(O).

## Supplementary Material

Crystal structure: contains datablock(s) I. DOI: 10.1107/S2056989019009812/lh5911sup1.cif

Structure factors: contains datablock(s) I. DOI: 10.1107/S2056989019009812/lh5911Isup2.hkl

CCDC reference: 1939397

Additional supporting information:  crystallographic information; 3D view; checkCIF report

## Figures and Tables

**Table 1:** Selected bond lengths (Å)

Cu1—O8^i^	1.935 (3)	Cu3—N5	1.974 (3)
Cu1—N1	1.980 (3)	Cu3—N7	1.982 (3)
Cu1—N8	1.993 (3)	Cu3—O10^iii^	1.990 (3)
Cu1—O1	2.022 (2)	Cu3—O1	2.039 (2)
Cu1—O2	2.182 (3)	Cu3—O4	2.182 (3)
Cu2—O6^ii^	1.954 (3)	Se1—O5	1.632 (3)
Cu2—N2	1.979 (3)	Se1—O2	1.637 (3)
Cu2—N4	1.986 (3)	Se1—O3	1.645 (3)
Cu2—O1	2.039 (3)	Se1—O4	1.649 (3)
Cu2—O3	2.146 (3)		

Symmetry codes: (i) 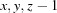; (ii) 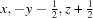; (iii) 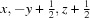.

**Table 2:** Hydrogen-bond geometry (Å, °)

*D*—H⋯*A*	*D*—H	H⋯*A*	*D*⋯*A*	*D*—H⋯*A*
O1—H1*O*⋯O10^iv^	0.85	2.01	2.811 (4)	156
O1*W*—H1*W*⋯O11	0.85	2.11	2.940 (5)	166
O1*W*—H2*W*⋯O4	0.85	1.93	2.767 (4)	168
O2*W*—H3*W*⋯O9^v^	0.85	1.95	2.798 (5)	178
O2*W*—H4*W*⋯O1*W*	0.85	2.00	2.812 (6)	159
C1—H1⋯O5^vi^	0.94	2.58	3.346 (5)	139
C2—H2⋯O5^vii^	0.94	2.28	2.955 (5)	128
C5—H5⋯O3^viii^	0.94	2.39	2.941 (5)	117
C6—H6⋯O2*W* ^iii^	0.94	2.30	3.176 (6)	154
C7—H7*A*⋯O3^viii^	0.98	2.25	3.094 (6)	144
C7—H7*B*⋯O1*W* ^iii^	0.98	2.38	3.338 (5)	164
C9—H9⋯O7^ix^	0.94	2.25	3.067 (6)	144

Symmetry codes: (iii) 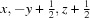; (iv) 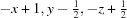; (v) 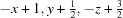; (vi) 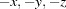; (vii) 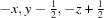; (viii) 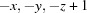; (ix) 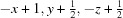.

**Table 3:** Experimental details

Crystal data	
Chemical formula	[Cu_3_(C_4_H_4_N_3_O_9_)_3_(SeO_4_)(OH)]·2H_2_O
*M*_r_	764.92
Crystal system, space group	Monoclinic, *P*2_1_/*c*
Temperature (K)	213
*a*, *b*, *c* (Å)	10.9403 (8), 17.5393 (15), 12.1289 (9)
β (°)	108.965 (8)
*V* (Å^3^)	2201.0 (3)
*Z*	4
Radiation type	Mo *K*α
μ (mm^−1^)	4.62
Crystal size (mm)	0.20 × 0.16 × 0.14

Data collection	
Diffractometer	Stoe Image plate diffraction system
Absorption correction	Numerical [*X-RED* (Stoe & Cie, 2001 ▸) and *X-SHAPE* (Stoe & Cie, 1999 ▸)]
*T*_min_, *T*_max_	0.405, 0.569
No. of measured, independent and observed [*I* > 2σ(*I*)] reflections	16928, 4681, 3306
*R*_int_	0.057
(sin θ/λ)_max_ (Å^−1^)	0.634

Refinement	
*R*[*F* ^2^ > 2σ(*F* ^2^)], *wR*(*F* ^2^), *S*	0.032, 0.072, 0.88
No. of reflections	4681
No. of parameters	343
H-atom treatment	H-atom parameters constrained
Δρ_max_, Δρ_min_ (e Å^−3^)	0.99, −0.64

Computer programs: *IPDS Software* (Stoe & Cie, 2000 ▸), *SHELXS97* (Sheldrick, 2008 ▸), *SHELXL2018* (Sheldrick, 2015 ▸), *DIAMOND* (Brandenburg, 1999 ▸) and *WinGX* (Farrugia, 2012 ▸).

## supplementary crystallographic information

### Crystal data

**Table:**

[Cu_3_(C_4_H_4_N_3_O_9_)_3_(SeO_4_)(OH)]·2H_2_O	*F*(000) = 1508
*M**_r_* = 764.92	*D*_x_ = 2.308 Mg m^−^^3^
Monoclinic, *P*2_1_/*c*	Mo *K*α radiation, λ = 0.71073 Å
*a* = 10.9403 (8) Å	Cell parameters from 8000 reflections
*b* = 17.5393 (15) Å	θ = 2.0–26.8°
*c* = 12.1289 (9) Å	µ = 4.62 mm^−^^1^
β = 108.965 (8)°	*T* = 213 K
*V* = 2201.0 (3) Å^3^	Prism, blue
*Z* = 4	0.20 × 0.16 × 0.14 mm

### Data collection

**Table:**

Stoe Image plate diffraction system diffractometer	3306 reflections with *I* > 2σ(*I*)
Radiation source: fine-focus sealed tube	*R*_int_ = 0.057
φ oscillation scans	θ_max_ = 26.8°, θ_min_ = 2.0°
Absorption correction: numerical [X-RED (Stoe & Cie, 2001) and X-SHAPE (Stoe & Cie, 1999)]	*h* = −13→13
*T*_min_ = 0.405, *T*_max_ = 0.569	*k* = −22→22
16928 measured reflections	*l* = −15→15
4681 independent reflections	

### Refinement

**Table:**

Refinement on *F*^2^	Primary atom site location: structure-invariant direct methods
Least-squares matrix: full	Secondary atom site location: difference Fourier map
*R*[*F*^2^ > 2σ(*F*^2^)] = 0.032	Hydrogen site location: inferred from neighbouring sites
*wR*(*F*^2^) = 0.072	H-atom parameters constrained
*S* = 0.88	*w* = 1/[σ^2^(*F*_o_^2^) + (0.0392*P*)^2^] where *P* = (*F*_o_^2^ + 2*F*_c_^2^)/3
4681 reflections	(Δ/σ)_max_ < 0.001
343 parameters	Δρ_max_ = 0.99 e Å^−^^3^
0 restraints	Δρ_min_ = −0.64 e Å^−^^3^

### Special details

**Table:**

Geometry. All esds (except the esd in the dihedral angle between two l.s. planes) are estimated using the full covariance matrix. The cell esds are taken into account individually in the estimation of esds in distances, angles and torsion angles; correlations between esds in cell parameters are only used when they are defined by crystal symmetry. An approximate (isotropic) treatment of cell esds is used for estimating esds involving l.s. planes.

### Fractional atomic coordinates and isotropic or equivalent isotropic displacement parameters (Å^2^)

**Table:**

	*x*	*y*	*z*	*U*_iso_*/*U*_eq_	
Cu1	0.27623 (5)	0.00328 (2)	0.16661 (4)	0.01311 (11)	
Cu2	0.17026 (5)	−0.07181 (2)	0.37617 (4)	0.01200 (11)	
Cu3	0.39077 (5)	0.07288 (3)	0.44294 (4)	0.01223 (11)	
Se1	0.08793 (4)	0.10530 (2)	0.27442 (3)	0.01415 (10)	
O1	0.3177 (3)	−0.01857 (14)	0.3386 (2)	0.0113 (5)	
H1O	0.378780	−0.050942	0.354877	0.017*	
O2	0.1203 (3)	0.08350 (16)	0.1556 (2)	0.0237 (7)	
O3	0.0502 (3)	0.02779 (15)	0.3325 (3)	0.0228 (7)	
O4	0.2182 (3)	0.14261 (15)	0.3694 (3)	0.0218 (7)	
O5	−0.0323 (3)	0.16534 (15)	0.2454 (3)	0.0231 (7)	
O6	0.0419 (3)	−0.36472 (15)	−0.0871 (2)	0.0174 (6)	
O7	0.2019 (3)	−0.28370 (17)	−0.0029 (3)	0.0301 (8)	
O8	0.2547 (3)	0.02330 (16)	1.0045 (2)	0.0253 (7)	
O9	0.3408 (4)	−0.0683 (2)	0.9233 (3)	0.0375 (9)	
O10	0.5155 (3)	0.36051 (14)	0.0589 (2)	0.0160 (6)	
O11	0.3676 (3)	0.28549 (17)	0.0946 (3)	0.0307 (8)	
N1	0.1686 (4)	−0.09003 (18)	0.1341 (3)	0.0148 (7)	
N2	0.1237 (4)	−0.11875 (18)	0.2195 (3)	0.0165 (7)	
N3	0.0471 (3)	−0.18786 (17)	0.0647 (3)	0.0139 (7)	
N4	0.2432 (3)	−0.03365 (18)	0.5386 (3)	0.0142 (7)	
N5	0.3327 (3)	0.02449 (18)	0.5642 (3)	0.0152 (7)	
N6	0.2564 (4)	0.01245 (19)	0.7085 (3)	0.0177 (8)	
N7	0.4666 (3)	0.10867 (18)	0.3243 (3)	0.0135 (7)	
N8	0.4102 (4)	0.08493 (17)	0.2103 (3)	0.0143 (7)	
N9	0.5346 (3)	0.18128 (17)	0.2111 (3)	0.0146 (7)	
C1	0.1208 (4)	−0.1327 (2)	0.0416 (4)	0.0174 (9)	
H1	0.135662	−0.125898	−0.029885	0.021*	
C2	0.0507 (4)	−0.1772 (2)	0.1748 (4)	0.0180 (9)	
H2	0.007193	−0.207386	0.214252	0.022*	
C3	−0.0143 (4)	−0.2514 (2)	−0.0127 (3)	0.0151 (8)	
H3A	−0.070577	−0.231321	−0.087360	0.018*	
H3B	−0.067585	−0.281162	0.022723	0.018*	
C4	0.0885 (4)	−0.3024 (2)	−0.0333 (3)	0.0153 (8)	
C5	0.1996 (4)	−0.0399 (2)	0.6262 (3)	0.0173 (9)	
H5	0.137619	−0.075582	0.631377	0.021*	
C6	0.3394 (5)	0.0504 (2)	0.6674 (4)	0.0209 (9)	
H6	0.394363	0.089864	0.707040	0.025*	
C7	0.2191 (5)	0.0356 (2)	0.8093 (3)	0.0196 (9)	
H7A	0.124993	0.030830	0.788261	0.024*	
H7B	0.240617	0.089592	0.824842	0.024*	
C8	0.2803 (4)	−0.0084 (2)	0.9205 (4)	0.0202 (9)	
C9	0.5423 (4)	0.1668 (2)	0.3220 (4)	0.0156 (8)	
H9	0.593603	0.193848	0.387670	0.019*	
C10	0.4526 (4)	0.1297 (2)	0.1453 (4)	0.0174 (9)	
H10	0.429103	0.126325	0.063676	0.021*	
C11	0.5883 (4)	0.2477 (2)	0.1676 (4)	0.0182 (9)	
H11A	0.635122	0.230577	0.115612	0.022*	
H11B	0.649085	0.275010	0.233175	0.022*	
C12	0.4785 (4)	0.3008 (2)	0.1017 (3)	0.0159 (9)	
O1W	0.3015 (4)	0.28114 (19)	0.3110 (4)	0.0441 (10)	
H1W	0.309086	0.277778	0.243576	0.066*	
H2W	0.264986	0.240868	0.323686	0.066*	
O2W	0.5427 (4)	0.3544 (2)	0.3669 (3)	0.0408 (9)	
H3W	0.576259	0.378437	0.430505	0.061*	
H4W	0.471669	0.335127	0.367685	0.061*	

### Atomic displacement parameters (Å^2^)

**Table:**

	*U*^11^	*U*^22^	*U*^33^	*U*^12^	*U*^13^	*U*^23^
Cu1	0.0193 (3)	0.0112 (2)	0.0091 (2)	−0.00358 (19)	0.0050 (2)	−0.00138 (17)
Cu2	0.0176 (3)	0.0096 (2)	0.0093 (2)	−0.00239 (19)	0.0050 (2)	−0.00079 (17)
Cu3	0.0169 (3)	0.0109 (2)	0.0091 (2)	−0.00224 (19)	0.0045 (2)	−0.00038 (17)
Se1	0.0155 (2)	0.01058 (17)	0.01497 (19)	0.00218 (16)	0.00297 (17)	0.00008 (15)
O1	0.0139 (16)	0.0091 (12)	0.0104 (12)	−0.0007 (10)	0.0033 (12)	−0.0019 (10)
O2	0.0289 (19)	0.0285 (16)	0.0131 (14)	0.0102 (13)	0.0061 (14)	0.0027 (12)
O3	0.0225 (18)	0.0143 (13)	0.0333 (17)	0.0021 (12)	0.0114 (15)	0.0066 (12)
O4	0.0208 (18)	0.0158 (14)	0.0243 (15)	−0.0005 (12)	0.0013 (14)	−0.0001 (12)
O5	0.0201 (18)	0.0172 (14)	0.0275 (16)	0.0101 (12)	0.0014 (15)	0.0007 (12)
O6	0.0208 (17)	0.0165 (13)	0.0147 (14)	0.0032 (12)	0.0056 (13)	−0.0039 (11)
O7	0.021 (2)	0.0212 (15)	0.046 (2)	0.0001 (14)	0.0079 (17)	−0.0058 (15)
O8	0.045 (2)	0.0250 (15)	0.0092 (13)	−0.0068 (14)	0.0130 (15)	−0.0017 (12)
O9	0.046 (2)	0.045 (2)	0.0257 (17)	0.0226 (18)	0.0176 (18)	0.0120 (16)
O10	0.0215 (17)	0.0116 (13)	0.0151 (14)	0.0002 (11)	0.0063 (13)	0.0023 (10)
O11	0.023 (2)	0.0256 (17)	0.041 (2)	−0.0006 (14)	0.0068 (17)	0.0117 (15)
N1	0.020 (2)	0.0161 (16)	0.0088 (15)	−0.0036 (13)	0.0059 (15)	−0.0001 (12)
N2	0.026 (2)	0.0129 (16)	0.0124 (16)	−0.0029 (14)	0.0086 (16)	−0.0006 (12)
N3	0.018 (2)	0.0099 (15)	0.0123 (16)	−0.0009 (13)	0.0032 (15)	−0.0016 (12)
N4	0.014 (2)	0.0145 (15)	0.0123 (16)	−0.0019 (13)	0.0023 (15)	−0.0024 (13)
N5	0.020 (2)	0.0157 (16)	0.0104 (16)	−0.0047 (14)	0.0050 (15)	−0.0008 (12)
N6	0.023 (2)	0.0201 (17)	0.0100 (16)	−0.0050 (14)	0.0057 (16)	−0.0009 (13)
N7	0.0153 (19)	0.0140 (15)	0.0102 (15)	−0.0015 (14)	0.0028 (15)	0.0001 (12)
N8	0.0177 (19)	0.0147 (16)	0.0096 (15)	−0.0005 (13)	0.0034 (15)	0.0002 (12)
N9	0.019 (2)	0.0107 (15)	0.0165 (17)	−0.0012 (13)	0.0089 (16)	0.0008 (12)
C1	0.023 (3)	0.0138 (18)	0.0154 (19)	−0.0007 (16)	0.0069 (19)	0.0008 (15)
C2	0.026 (3)	0.0131 (18)	0.017 (2)	−0.0056 (16)	0.010 (2)	−0.0036 (15)
C3	0.017 (2)	0.0120 (17)	0.0123 (19)	−0.0029 (15)	0.0001 (18)	−0.0027 (14)
C4	0.021 (3)	0.0128 (18)	0.0108 (18)	−0.0008 (16)	0.0037 (18)	0.0023 (14)
C5	0.020 (3)	0.021 (2)	0.0123 (19)	−0.0051 (17)	0.0077 (19)	−0.0011 (15)
C6	0.027 (3)	0.022 (2)	0.0140 (19)	−0.0078 (18)	0.007 (2)	0.0000 (16)
C7	0.025 (3)	0.022 (2)	0.0127 (19)	−0.0013 (18)	0.0076 (19)	−0.0029 (16)
C8	0.019 (2)	0.023 (2)	0.018 (2)	−0.0069 (18)	0.004 (2)	−0.0012 (17)
C9	0.018 (2)	0.0141 (18)	0.0146 (19)	0.0023 (16)	0.0048 (18)	−0.0006 (15)
C10	0.023 (3)	0.0156 (18)	0.016 (2)	−0.0018 (16)	0.009 (2)	0.0009 (15)
C11	0.021 (3)	0.0146 (19)	0.021 (2)	−0.0025 (17)	0.010 (2)	0.0057 (16)
C12	0.023 (3)	0.0129 (17)	0.0122 (19)	−0.0027 (16)	0.0061 (19)	0.0006 (14)
O1W	0.053 (3)	0.0231 (17)	0.064 (3)	−0.0041 (17)	0.029 (2)	0.0046 (17)
O2W	0.037 (2)	0.054 (2)	0.0280 (19)	−0.0025 (18)	0.0055 (18)	−0.0045 (17)

### Geometric parameters (Å, º)

**Table:**

Cu1—O8^i^	1.935 (3)	N4—C5	1.303 (5)
Cu1—N1	1.980 (3)	N4—N5	1.377 (4)
Cu1—N8	1.993 (3)	N5—C6	1.311 (5)
Cu1—O1	2.022 (2)	N6—C6	1.346 (5)
Cu1—O2	2.182 (3)	N6—C5	1.349 (5)
Cu2—O6^ii^	1.954 (3)	N6—C7	1.466 (5)
Cu2—N2	1.979 (3)	N7—C9	1.320 (5)
Cu2—N4	1.986 (3)	N7—N8	1.382 (4)
Cu2—O1	2.039 (3)	N8—C10	1.301 (5)
Cu2—O3	2.146 (3)	N9—C10	1.339 (5)
Cu3—N5	1.974 (3)	N9—C9	1.345 (5)
Cu3—N7	1.982 (3)	N9—C11	1.478 (5)
Cu3—O10^iii^	1.990 (3)	C1—H1	0.9400
Cu3—O1	2.039 (2)	C2—H2	0.9400
Cu3—O4	2.182 (3)	C3—C4	1.520 (6)
Se1—O5	1.632 (3)	C3—H3A	0.9800
Se1—O2	1.637 (3)	C3—H3B	0.9800
Se1—O3	1.645 (3)	C5—H5	0.9400
Se1—O4	1.649 (3)	C6—H6	0.9400
O1—H1O	0.8500	C7—C8	1.509 (6)
O6—C4	1.289 (5)	C7—H7A	0.9800
O7—C4	1.219 (5)	C7—H7B	0.9800
O8—C8	1.270 (5)	C9—H9	0.9400
O9—C8	1.235 (5)	C10—H10	0.9400
O10—C12	1.291 (4)	C11—C12	1.525 (6)
O11—C12	1.218 (5)	C11—H11A	0.9800
N1—C1	1.308 (5)	C11—H11B	0.9800
N1—N2	1.379 (4)	O1W—H1W	0.8500
N2—C2	1.304 (5)	O1W—H2W	0.8500
N3—C2	1.337 (5)	O2W—H3W	0.8500
N3—C1	1.346 (5)	O2W—H4W	0.8500
N3—C3	1.471 (5)		
			
O8^i^—Cu1—N1	94.55 (13)	C6—N5—Cu3	129.1 (3)
O8^i^—Cu1—N8	88.65 (13)	N4—N5—Cu3	121.9 (2)
N1—Cu1—N8	170.10 (14)	C6—N6—C5	105.5 (3)
O8^i^—Cu1—O1	174.35 (13)	C6—N6—C7	125.2 (3)
N1—Cu1—O1	88.18 (11)	C5—N6—C7	128.2 (3)
N8—Cu1—O1	87.86 (12)	C9—N7—N8	107.0 (3)
O8^i^—Cu1—O2	89.20 (12)	C9—N7—Cu3	132.3 (3)
N1—Cu1—O2	96.79 (13)	N8—N7—Cu3	118.3 (2)
N8—Cu1—O2	92.62 (13)	C10—N8—N7	107.0 (3)
O1—Cu1—O2	95.40 (10)	C10—N8—Cu1	130.4 (3)
O6^ii^—Cu2—N2	90.12 (12)	N7—N8—Cu1	122.2 (2)
O6^ii^—Cu2—N4	93.00 (12)	C10—N9—C9	106.3 (3)
N2—Cu2—N4	170.93 (15)	C10—N9—C11	125.9 (3)
O6^ii^—Cu2—O1	172.41 (11)	C9—N9—C11	127.1 (3)
N2—Cu2—O1	87.23 (12)	N1—C1—N3	109.4 (3)
N4—Cu2—O1	88.59 (12)	N1—C1—H1	125.3
O6^ii^—Cu2—O3	95.29 (11)	N3—C1—H1	125.3
N2—Cu2—O3	98.97 (13)	N2—C2—N3	109.9 (3)
N4—Cu2—O3	89.24 (13)	N2—C2—H2	125.0
O1—Cu2—O3	92.15 (11)	N3—C2—H2	125.0
N5—Cu3—N7	171.64 (14)	N3—C3—C4	110.0 (3)
N5—Cu3—O10^iii^	92.21 (12)	N3—C3—H3A	109.7
N7—Cu3—O10^iii^	88.56 (12)	C4—C3—H3A	109.7
N5—Cu3—O1	88.15 (12)	N3—C3—H3B	109.7
N7—Cu3—O1	88.32 (12)	C4—C3—H3B	109.7
O10^iii^—Cu3—O1	160.30 (11)	H3A—C3—H3B	108.2
N5—Cu3—O4	95.76 (13)	O7—C4—O6	125.3 (4)
N7—Cu3—O4	92.07 (13)	O7—C4—C3	121.7 (3)
O10^iii^—Cu3—O4	105.28 (11)	O6—C4—C3	112.9 (4)
O1—Cu3—O4	94.27 (11)	N4—C5—N6	110.1 (4)
O5—Se1—O2	110.53 (15)	N4—C5—H5	125.0
O5—Se1—O3	109.17 (15)	N6—C5—H5	125.0
O2—Se1—O3	109.91 (15)	N5—C6—N6	110.3 (4)
O5—Se1—O4	110.35 (15)	N5—C6—H6	124.9
O2—Se1—O4	108.82 (16)	N6—C6—H6	124.9
O3—Se1—O4	108.01 (15)	N6—C7—C8	116.2 (4)
Cu1—O1—Cu3	113.72 (11)	N6—C7—H7A	108.2
Cu1—O1—Cu2	112.88 (13)	C8—C7—H7A	108.2
Cu3—O1—Cu2	113.63 (11)	N6—C7—H7B	108.2
Cu1—O1—H1O	105.2	C8—C7—H7B	108.2
Cu3—O1—H1O	105.1	H7A—C7—H7B	107.4
Cu2—O1—H1O	105.2	O9—C8—O8	127.3 (4)
Se1—O2—Cu1	119.02 (15)	O9—C8—C7	122.5 (4)
Se1—O3—Cu2	124.02 (16)	O8—C8—C7	110.1 (4)
Se1—O4—Cu3	119.98 (15)	N7—C9—N9	109.3 (4)
C4—O6—Cu2^iv^	113.7 (3)	N7—C9—H9	125.3
C8—O8—Cu1^v^	138.6 (3)	N9—C9—H9	125.3
C12—O10—Cu3^vi^	121.8 (3)	N8—C10—N9	110.4 (4)
C1—N1—N2	107.1 (3)	N8—C10—H10	124.8
C1—N1—Cu1	133.6 (3)	N9—C10—H10	124.8
N2—N1—Cu1	119.1 (2)	N9—C11—C12	109.4 (3)
C2—N2—N1	107.1 (3)	N9—C11—H11A	109.8
C2—N2—Cu2	131.1 (3)	C12—C11—H11A	109.8
N1—N2—Cu2	121.7 (2)	N9—C11—H11B	109.8
C2—N3—C1	106.4 (3)	C12—C11—H11B	109.8
C2—N3—C3	127.1 (3)	H11A—C11—H11B	108.2
C1—N3—C3	126.3 (3)	O11—C12—O10	125.9 (4)
C5—N4—N5	107.5 (3)	O11—C12—C11	120.0 (3)
C5—N4—Cu2	130.2 (3)	O10—C12—C11	114.1 (4)
N5—N4—Cu2	120.0 (2)	H1W—O1W—H2W	108.4
C6—N5—N4	106.7 (3)	H3W—O2W—H4W	108.4
			
O5—Se1—O2—Cu1	−179.53 (17)	Cu2^iv^—O6—C4—C3	176.2 (2)
O3—Se1—O2—Cu1	−59.0 (2)	N3—C3—C4—O7	−10.4 (5)
O4—Se1—O2—Cu1	59.1 (2)	N3—C3—C4—O6	170.9 (3)
O5—Se1—O3—Cu2	175.78 (18)	N5—N4—C5—N6	0.4 (5)
O2—Se1—O3—Cu2	54.4 (2)	Cu2—N4—C5—N6	−161.7 (3)
O4—Se1—O3—Cu2	−64.2 (2)	C6—N6—C5—N4	−1.1 (5)
O5—Se1—O4—Cu3	172.90 (16)	C7—N6—C5—N4	167.1 (4)
O2—Se1—O4—Cu3	−65.7 (2)	N4—N5—C6—N6	−1.2 (5)
O3—Se1—O4—Cu3	53.6 (2)	Cu3—N5—C6—N6	161.3 (3)
C1—N1—N2—C2	−0.1 (5)	C5—N6—C6—N5	1.4 (5)
Cu1—N1—N2—C2	176.8 (3)	C7—N6—C6—N5	−167.2 (4)
C1—N1—N2—Cu2	−179.7 (3)	C6—N6—C7—C8	−103.6 (5)
Cu1—N1—N2—Cu2	−2.8 (4)	C5—N6—C7—C8	90.4 (5)
C5—N4—N5—C6	0.4 (5)	Cu1^v^—O8—C8—O9	−4.3 (8)
Cu2—N4—N5—C6	164.7 (3)	Cu1^v^—O8—C8—C7	172.5 (3)
C5—N4—N5—Cu3	−163.6 (3)	N6—C7—C8—O9	−11.9 (6)
Cu2—N4—N5—Cu3	0.7 (4)	N6—C7—C8—O8	171.1 (4)
C9—N7—N8—C10	−0.7 (4)	N8—N7—C9—N9	0.8 (4)
Cu3—N7—N8—C10	163.8 (3)	Cu3—N7—C9—N9	−160.7 (3)
C9—N7—N8—Cu1	−175.1 (3)	C10—N9—C9—N7	−0.6 (5)
Cu3—N7—N8—Cu1	−10.5 (4)	C11—N9—C9—N7	170.5 (4)
N2—N1—C1—N3	−0.1 (5)	N7—N8—C10—N9	0.4 (5)
Cu1—N1—C1—N3	−176.4 (3)	Cu1—N8—C10—N9	174.1 (3)
C2—N3—C1—N1	0.4 (5)	C9—N9—C10—N8	0.1 (5)
C3—N3—C1—N1	−174.2 (4)	C11—N9—C10—N8	−171.1 (4)
N1—N2—C2—N3	0.4 (5)	C10—N9—C11—C12	62.3 (5)
Cu2—N2—C2—N3	179.8 (3)	C9—N9—C11—C12	−107.2 (4)
C1—N3—C2—N2	−0.4 (5)	Cu3^vi^—O10—C12—O11	−13.8 (6)
C3—N3—C2—N2	174.0 (4)	Cu3^vi^—O10—C12—C11	167.2 (2)
C2—N3—C3—C4	−110.0 (5)	N9—C11—C12—O11	1.7 (5)
C1—N3—C3—C4	63.4 (5)	N9—C11—C12—O10	−179.2 (3)
Cu2^iv^—O6—C4—O7	−2.5 (5)		

Symmetry codes: (i) *x*, *y*, *z*−1; (ii) *x*, −*y*−1/2, *z*+1/2; (iii) *x*, −*y*+1/2, *z*+1/2; (iv) *x*, −*y*−1/2, *z*−1/2; (v) *x*, *y*, *z*+1; (vi) *x*, −*y*+1/2, *z*−1/2.

### Hydrogen-bond geometry (Å, º)

**Table:**

*D*—H···*A*	*D*—H	H···*A*	*D*···*A*	*D*—H···*A*
O1—H1*O*···O10^vii^	0.85	2.01	2.811 (4)	156
O1*W*—H1*W*···O11	0.85	2.11	2.940 (5)	166
O1*W*—H2*W*···O4	0.85	1.93	2.767 (4)	168
O2*W*—H3*W*···O9^viii^	0.85	1.95	2.798 (5)	178
O2*W*—H4*W*···O1*W*	0.85	2.00	2.812 (6)	159
C1—H1···O5^ix^	0.94	2.58	3.346 (5)	139
C2—H2···O5^x^	0.94	2.28	2.955 (5)	128
C5—H5···O3^xi^	0.94	2.39	2.941 (5)	117
C6—H6···O2*W*^iii^	0.94	2.30	3.176 (6)	154
C7—H7*A*···O3^xi^	0.98	2.25	3.094 (6)	144
C7—H7*B*···O1*W*^iii^	0.98	2.38	3.338 (5)	164
C9—H9···O7^xii^	0.94	2.25	3.067 (6)	144

Symmetry codes: (iii) *x*, −*y*+1/2, *z*+1/2; (vii) −*x*+1, *y*−1/2, −*z*+1/2; (viii) −*x*+1, *y*+1/2, −*z*+3/2; (ix) −*x*, −*y*, −*z*; (x) −*x*, *y*−1/2, −*z*+1/2; (xi) −*x*, −*y*, −*z*+1; (xii) −*x*+1, *y*+1/2, −*z*+1/2.
